# Imaging Findings of Mirizzi Syndrome Associated With Acalculous Cholecystitis: A Case Report

**DOI:** 10.7759/cureus.78261

**Published:** 2025-01-30

**Authors:** David Martinez Juarez, Omar Santos Moreno, Omar Gomez Monterrosas, David Hernandez Arango, Florencio Ortiz Santos

**Affiliations:** 1 Department of Radiology, Christus Muguerza Hospital Betania, Puebla, MEX; 2 Department of Radiology, Hospital de Especialidades 5 de Mayo, Instituto de Seguridad y Servicios Sociales de los Trabajadores al Servicio de los Poderes del Estado de Puebla (ISSSTEP), Puebla, MEX; 3 Department of Cardiology, Hospital Angeles Puebla, Puebla, MEX

**Keywords:** alithiasic mirizzi syndrome, case report, cholecystitis, mirizzi syndrome, mrcp, mri

## Abstract

Right upper quadrant pain is one of the most frequent reasons for consultations in the emergency room. Gallbladder pathology is among the most common etiologies and can include cholecystitis, cholelithiasis, choledocholithiasis, and cholangitis, among others. Mirizzi syndrome is a complication that manifests as hepatobiliary dysfunction due to a gallstone causing extrinsic compression of the common bile duct. However, acute cholecystitis can externally obstruct the common bile duct and mimic Mirizzi syndrome in the absence of a causative gallstone. The relevance of this syndrome lies in its timely imaging diagnosis, allowing physicians to rule out other biliary pathologies and to identify its different presentations before surgical intervention. A 72-year-old male presented with abdominal pain in the right upper quadrant for the past four days. The pain persisted, prompting his admission to the emergency department. On examination, he reported abdominal pain with a positive Murphy sign. Initial abdominal ultrasound revealed dilation of the common hepatic duct, hydrocholecyst, and thickening of the gallbladder wall. Abdominal computed tomography (CT) suggested extrinsic compression of the extrahepatic bile duct by the gallbladder. Laboratory tests revealed significant leukocytosis with neutrophilia, and inflammatory markers, including C-reactive protein and erythrocyte sedimentation rate, were elevated. Liver function tests, however, remained within normal limits, with only a slight elevation in gamma-glutamyl transferase (GGT). Magnetic resonance imaging with gadolinium ruled out neoplasia of the pancreas. A magnetic resonance cholangiopancreatography (MRCP) confirmed dilation of the extrahepatic bile duct caused by extrinsic compression from the gallbladder infundibulum at the hepatic hilum and proximal common bile duct, along with slight dilation of the intrahepatic bile duct. Hydrocholecyst and gallbladder wall thickening with signal changes due to edema were also observed. These findings confirmed an extremely rare alithiasic presentation of type I Mirizzi syndrome. The patient was offered a cholecystectomy; however, he declined surgical treatment. Conservative management was pursued, and a follow-up ultrasound performed two days later showed a significant reduction in gallbladder volume, correlating with clinical improvement.

## Introduction

Mirizzi syndrome, a rare complication of biliary tract obstruction due to gallstones, has an incidence ranging from approximately 0.1% to 2% of patients with biliary pathologies [1]. In Latin America, its prevalence varies from 4.7% to 5.7%, dropping to 1% in northern countries [2]. This condition is characterized by the impaction of one or more calculi in Hartmann's pouch or the cystic duct, leading to compression and extrinsic obstruction of the common hepatic duct. Mirizzi syndrome presents similarly to acute cholecystitis but with the presence of jaundice in addition to biliary duct obstruction [3,4].

Clinically, this syndrome manifests as abdominal pain, predominantly in the right hypochondrium, accompanied by intermittent obstructive pattern jaundice due to biliary duct obstruction. Additionally, nausea and vomiting may be present [5].

As a diagnostic imaging tool, abdominal ultrasound is the first approach; however, magnetic resonance cholangiopancreatography (MRCP) is considered the most sensitive (96%) and specific (94%) imaging method and is the gold standard for preoperative diagnosis and anatomical visualization before surgery [2]. This approach facilitates better determination of surgical treatment and can determine whether cholecystectomy or endoscopic retrograde cholangiopancreatography (ERCP) to decompress the bile duct is indicated [6]. In our case, we emphasize the importance of preoperative diagnosis through MRCP in a male patient who presented with lithiasis-free Mirizzi syndrome.

## Case presentation

A 72-year-old male, presented with abdominal pain, rated 7/10 on the visual analog scale (VAS) localized to the right upper quadrant, radiating to the right hypochondrium and the right shoulder. The patient had been experiencing pain for four days prior to evaluation. Despite self-medicating with analgesics, there was no improvement. On the contrary, he developed nausea, bilious vomiting, and worsening pain, prompting him to seek urgent medical attention. On examination, the patient reported moderate pain with stable vital signs. A positive Murphy sign was noted, but there were no signs of peritoneal irritation.

The patient reported a history of post-traumatic stress disorder, managed with 400 mg of oral quetiapine daily. He also had a history of pulmonary thromboembolism in December 2023, treated with 5 mg of apixaban every 12 hours. Five years earlier, the patient had undergone cystoscopy, bilateral inguinal hernioplasty, and diagnostic cardiac catheterization. No significant weight loss was reported. There were no other notable personal clinical histories. However, the patient had an active history of smoking and alcohol consumption.

On physical examination, the patient had a heart rate of 80 beats per minute, respiratory rate of 12 breaths per minute, blood pressure of 110/80 mmHg, and oxygen saturation of 98%. The abdomen was soft and depressible, with normal peristalsis. However, tenderness was noted on palpation in the mid-abdomen, accompanied by a positive Murphy sign but no evidence of peritoneal irritation. The remainder of the physical examination revealed no significant abnormalities.

Upon admission, laboratory tests (Table 1) revealed significant leukocytosis with neutrophilia. Inflammatory markers, including C-reactive protein and erythrocyte sedimentation rate, were elevated. However, liver function tests remained within normal limits, with only a slight elevation in gamma-glutamyl transferase (GGT).

**Table 1:** Summary of laboratory findings

Laboratory Test	Result	Reference
Total Bilirubin	0.63 mg/dL	0.1-1.2 mg/dL
Direct Bilirubin	0.4 mg/dL	0-0.4 mg/dL
Indirect Bilirubin	0.23 mg/dL	0.1-0.8 mg/dL
Aspartate Aminotransferase (AST)	29 U/L	10-40 U/L
Alanine Aminotransferase (ALT)	18 U/L	7-56 U/L
Alkaline Phosphatase	64 U/L	44-147 U/L
Lactate Dehydrogenase (LDH)	160 U/L	140-280 U/L
Gamma-Glutamyl Transferase (GGT)	70 U/L	0-60 U/L
White Blood Cell Count (WBC)	12.76 k/µL	4.5-11.0 k/µL
Neutrophils (%)	87%	40%-70%
C-Reactive Protein (CRP)	76.96 mg/L	< 10 mg/L
Hemoglobin	14.0 g/dL	14.0-17.5 g/dL
Platelets	196x10³/µL	150-450 x10³/µL

**Figure 1:**
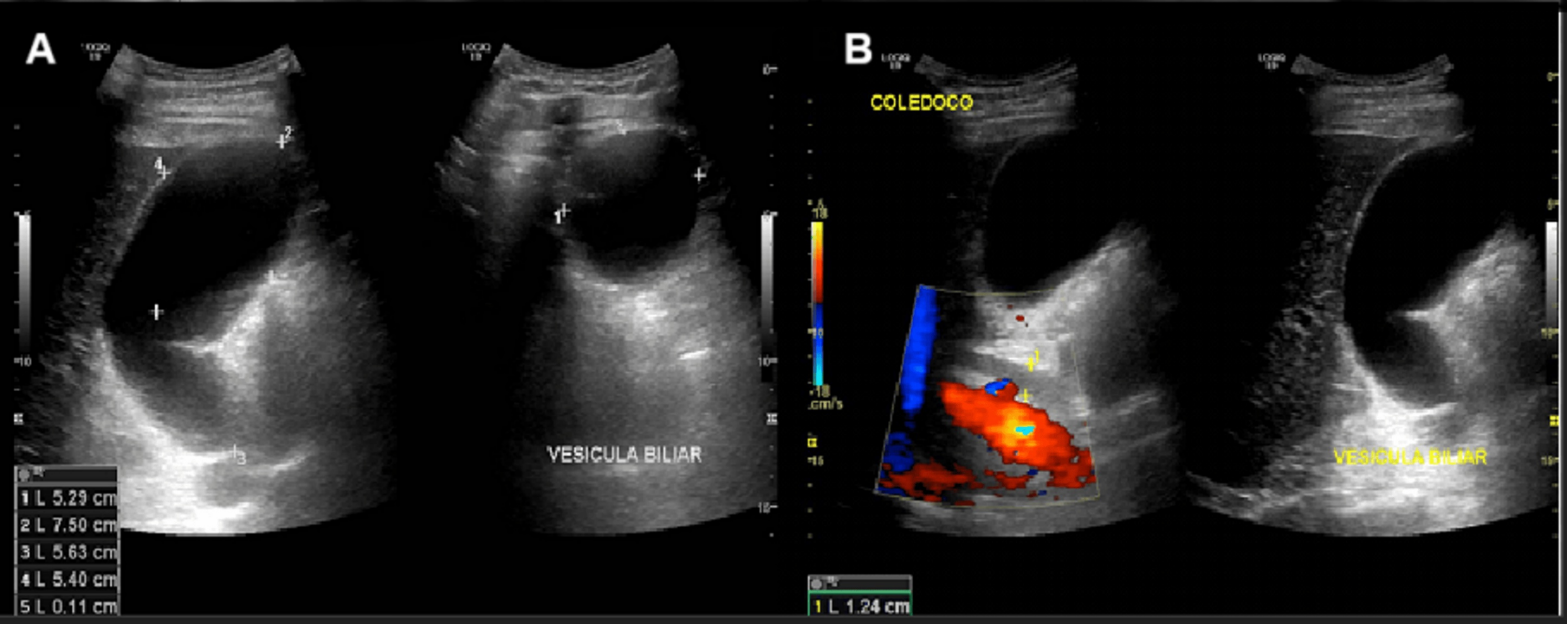
Abdominal ultrasoud findings A) Gallbladder hydrops with a calculated volume of 191 cc. B) Dilation of the common bile duct measuring 12 mm.

**Figure 2:**
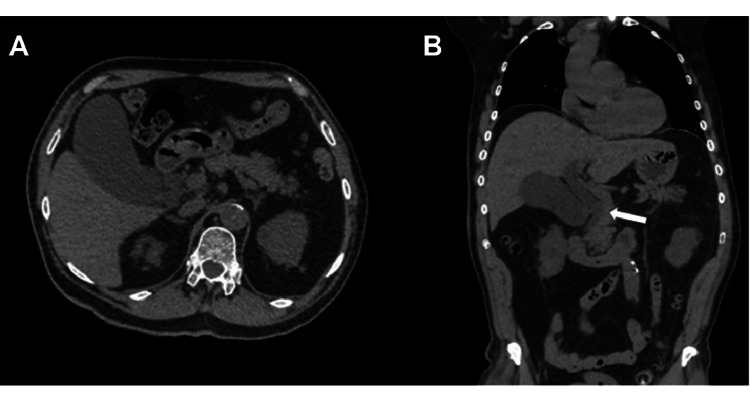
Abdominal CT findings A) Axial CT image demostrating gallbladder hydrops. B) Coronal CT image evidencing compression of the common bile duct by the infundibulum and retrograde dilation of the duct.

**Figure 3:**
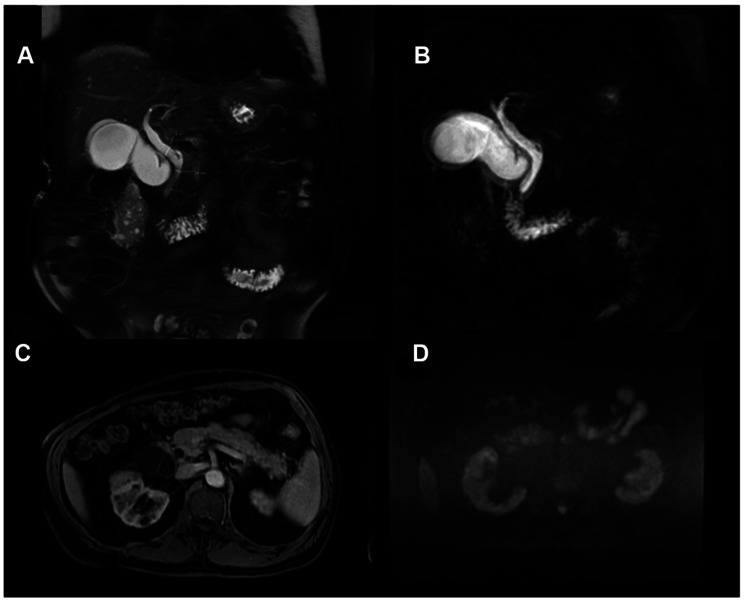
Contrast-enhanced abdominal MRI A) T2-weighted fat-saturated coronal MR image demonstrating dilation of the common hepatic duct (CHD) up to 12mm. B) 3D MRCP image confirming proximal common bile duct dilation caused by extrinsic compression from the gallbladder infundibulum. The pancreatic duct appears normal. An anatomical variant is observed, with the cystic duct inserting posteriorly and medially into the extrahepatic bile duct. C) T1-weighted contrast-enhanced image and D) diffusion-weighted imaging (DWI) sequence showing no evidence of neoplasm at the head of the pancreas.

The initial abdominal ultrasound revealed a 12 mm dilation of the common hepatic duct (Figure 1, images A and B) and a distended gallbladder (without stones) with a calculated volume of 191 cc. These findings were further evaluated with an abdominal computed tomography scan (Figure 2, images A and B), which identified dilation of the common hepatic duct caused by apparent extrinsic compression of the extrahepatic bile duct by the gallbladder. Contrast-enhanced magnetic resonance imaging (Figure 3, image C) ruled out neoplasms at the level of the pancreatic head, while diffusion-weighted imaging (Figure 3, image D) confirmed the absence of restricted diffusion zones. MRCP (Figure 3, images A and B) confirmed dilation of the common hepatic bile duct and mild dilation of the intrahepatic bile duct secondary to extrinsic compression of the gallbladder infundibulum at the hepatic hilum and proximal common bile duct. Hydrocholecyst and gallbladder wall thickening (4 mm), consistent with edema-related signal changes, were also observed. Additionally, an anatomical variant of the cystic duct with a medial insertion into the bile duct was identified.

**Figure 4:**
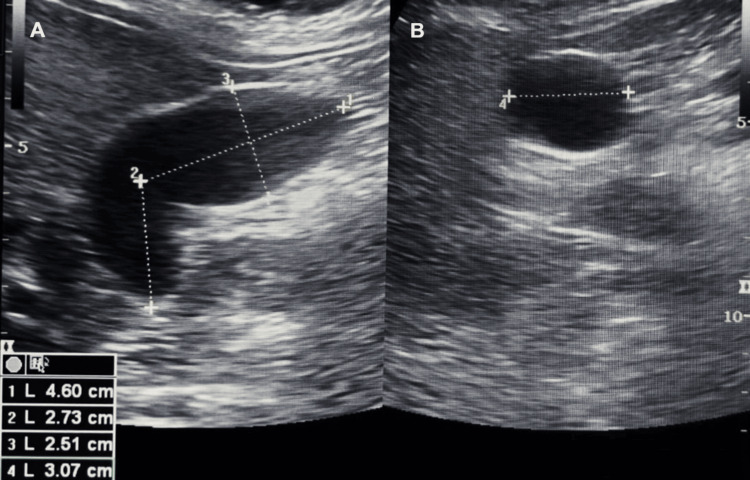
Follow-up abdominal ultrasound Images A) and B) confirming a reduction in gallbladder volume (29 cc) and showing no compression of the common bile duct.

The patient was diagnosed with lithiasis-free Mirizzi syndrome type I, a rare presentation characterized by extrinsic compression of the common bile duct by the gallbladder infundibulum in the absence of gallstones. Medical treatment was initiated with meropenem (1 g every eight hours), buprenorphine (900 mg per day), metronidazole (500 mg every eight hours), and paracetamol (1 g every eight hours), leading to symptom improvement. A follow-up abdominal ultrasound revealed a reduction in gallbladder volume in both longitudinal and transverse views (Figure 4, images A and B), consistent with clinical improvement. Surgical treatment with cholecystectomy was offered but declined by the patient, who opted for conservative management instead.

## Discussion

Right upper quadrant pain is one of the most frequent reasons for consultations in the emergency room. Gallbladder pathology is among the most common etiologies and can include cholecystitis, cholelithiasis, choledocholithiasis, and cholangitis, among others [7]. Mirizzi syndrome is a complication that manifests as hepatobiliary dysfunction due to a gallstone causing extrinsic compression of the common bile duct. However, acute cholecystitis can externally obstruct the common bile duct and mimic Mirizzi syndrome in the absence of a causative gallstone. The relevance of this syndrome lies in its timely imaging diagnosis, allowing physicians to rule out other biliary pathologies and to identify its different presentations before surgical intervention.

The diagnosis of Mirizzi syndrome presents challenges because of its low incidence and clinical presentation, which often resembles that of cholelithiasis [3,4]. Thus, it is common for it to go unnoticed or to be diagnosed after ruling out other biliary pathologies or during surgery. According to reported cases [8,9], some patients are asymptomatic, and their diagnosis is incidental during medical check-ups. Others present typical symptoms of acute cholecystitis, as in this case, which is the most common presentation in documented cases and the predominant reason for emergency department visits. In isolated cases, more complex symptoms, such as jaundice, fever, or even debut with an oncological process, may occur.

Imaging studies are fundamental for both the diagnosis and surgical planning of Mirizzi syndrome [10]. The classification presented in Table 2 provides essential criteria for differentiating the various types of this condition.

**Table 2:** Mirizzi classification Source: Ref [10]

Type	Description
Type I	External compression of the common bile duct
Ia	The common bile duct runs alongside a long cystic duct
Ib	The common bile duct runs alongside a short cystic duct
Type II	Presence of gallbladder-biliary fistula with a diameter <1/3 of the common bile duct
IIa	Gallbladder-biliary fistula with a diameter <50% of the common bile duct
IIb	Gallbladder-biliary fistula with a diameter >50% of the common bile duct
Type III	Presence of gallbladder-biliary fistula with a diameter >2/3 of the common bile duct
IIIa	Without gallstone ileus
IIIb	With gallstone ileus
Type IV	Complete destruction of the common bile duct wall
Type V	Formation of a gallbladder-enteric fistula
Va	Cholecystectomy and closure of the fistula
Vb	Break-up of the gallstone ileus (enterolithotomy) and cholecystectomy once the patient recovers

Due to Mirizzi syndrome’s clinical similarity to that of cholelithiasis, abdominal ultrasound is considered the initial diagnostic method due to its high sensitivity in detecting cholelithiasis. However, its specificity in diagnosing Mirizzi syndrome is limited [10]. Common ultrasound findings include common hepatic duct and intrahepatic bile duct dilation, gallbladder wall thickening, and the presence of stones embedded in Hartmann's pouch. Notably, in this case report, hydrocholecyst and dilation of the common bile duct were observed without gallbladder lithiasis. At this point, owing to the clinical and imaging findings (positive Morphy's sign, leukocytosis, elevated CRP, thickened-walled vesicles, and hydrocholecyst), the Tokyo criteria for cholecystitis were met, which constituted an extremely atypical presentation of Mirizzi syndrome.

Given the complexity of the diagnosis and the diversity of symptoms, CT scans are frequently used for diagnosis, although their sensitivity to Mirizzi syndrome is poor (42%). CT is usually requested because of suspicion of neoplastic processes [11]. The most frequent findings reported by CT are intra- and extrahepatic bile duct dilation, wall edema, and pericholecystic fluid. In this case, CT revealed hydrocholecyst with intra- and extrahepatic bile duct dilation, prompting further diagnostic studies.

ERCP is the gold standard for the diagnosis of biliary and pancreatic obstructive pathologies, but its use may be limited due to inherent complications. As an alternative, MRCP stands out for its high sensitivity of 96% and specificity of 94%, making it an excellent diagnostic test for Mirizzi syndrome [5]. The plethora of signs and symptoms involving Mirizzi syndrome include a distended gallbladder with homogeneous content, perihepatic and pericholecystic fluid, dilation of the hepatic duct, and slight dilation of the intrahepatic ducts with and without filling defects. Taken together, these produce a mass effect on the bile duct.

In accordance with the clinically available evidence and the patient’s findings presented (compression from the infundibulum over the common bile duct without an immediate obstructive cause in the biliary tract and a normal cystic duct with an anatomical variant (posterior spiral and medial insertion) observed in the MRCP sequence, the diagnostic impression of alithiasic Mirizzi syndrome type I was attained [12].

Notably, MRCP not only provides an accurate diagnosis but is also capable of detecting the presence of gallstones, sites of stenosis in the bile ducts, and even trajectories of biliary fistulas. This information is crucial for proper classification [3].

## Conclusions

This case highlights the unique and rare alithiasic presentation of Mirizzi syndrome, emphasizing the challenges in diagnosing this condition due to its clinical similarity to more common biliary pathologies such as acute cholecystitis or choledocholithiasis. The integration of advanced imaging modalities, particularly MRCP, proved invaluable in characterizing the anatomical and pathological features, enabling an accurate preoperative diagnosis. This underscores the importance of MRCP not only as a diagnostic gold standard but also as a critical tool for surgical planning, particularly in atypical cases where gallstones are absent. Furthermore, this case also highlights the successful use of conservative management as an alternative approach in select patients, underscoring the importance of individualized treatment strategies based on clinical presentation and patient preferences.
